# Supplementary material for: Molecular Mechanisms Underlying the Anti-Inflammatory Properties of (R)-(-)-Carvone: Potential Roles of JNK1, Nrf2 and NF-κB
Source: Pharmaceutics. 2023 Jan 11;15(1):249. doi: 10.3390/pharmaceutics15010249 (PMC9865770; doi:10.3390/pharmaceutics15010249)
Supplement: Supplementary file 1 [file pharmaceutics-15-00249-s001.zip › pharmaceutics-2106963-supplementary.pdf]

Supporting Information File to:

Molecular mechanisms underlying the anti-inflammatory properties of (R)-(-)-carvone: potential role of JNK1, Nrf2 and NF-κB

Supplementary Tables S1

Supplementary raw data to Figure 2C

p-JNK1/JNK ratios and respective p values

	N1		N2		N3		N4		p-value	
	**PI/LC**	**NR**	**PI/LC**	**NR**	**PI/LC**	**NR**	**PI/LC**	**NR**	***vs.* LPS**	***vs.* Ctrl**
**Ctrl**	0.02	0.04	0.02	0.05	0.02	0.03	0.04	0.04	<0.0001	------
**LPS**	0.53	1.0	0.33	1.0	0.66	1.0	1.10	1.0	------	<0.0001
**(R)-carv. + LPS**	0.16	0.30	0.05	0.15	0.04	0.06	0.52	0.48	0.0063	0.1711

PI/LC: Protein of Interest/Loading Control; **NR:** Normalized Ratio

p-JNK2/JNK ratios and respective p values

	N1		N2		N3		N4		p-value	
	**PI/LC**	**NR**	**PI/LC**	**NR**	**PI/LC**	**NR**	**PI/LC**	**NR**	***vs.* LPS**	***vs.* Ctrl**
**Ctrl**	0.01	0.04	0.01	0.04	0.02	0.02	0.04	0.02	<0.0001	------
**LPS**	0.35	1.0	0.27	1.0	0.92	1.0	1.74	1.0	------	<0.0001
**(R)-carv. + LPS**	0.29	0.83	0.10	0.38	0.31	0.34	1.59	0.91	0.1296	0.0478

PI/LC: Protein of Interest/Loading Control; **NR:** Normalized Ratio

p-JNK3/JNK ratios and respective p values

	N1		N2		N3		N4		p-value	
	**PI/LC**	**NR**	**PI/LC**	**NR**	**PI/LC**	**NR**	**PI/LC**	**NR**	***vs.* LPS**	***vs.* Ctrl**
**Ctrl**	0.01	0.03	0.01	0.02	0.03	0.02	0.05	0.02	<0.0001	------
**LPS**	0.48	1.0	0.41	1.0	1.29	1.0	2.66	1.0	------	<0.0001
**(R)-carv. + LPS**	0.38	0.80	0.12	0.31	0.36	0.28	2.23	0.84	0.0999	0.0619

PI/LC: Protein of Interest/Loading Control; **NR:** Normalized Ratio

Supplementary Table S2

Supplementary raw data to Figure 6A

Ac-p65(Lys310)/Lamin B1 ratios and respective p values

	N1		N2		N3		p-value	
	**PI/LC**	**NR**	**PI/LC**	**NR**	**PI/LC**	**NR**	***vs.* LPS**	***vs.* Ctrl**
**Ctrl**	0.012	1.13	0.009	0.65	0.0013	0.54	0.4855	------
**LPS**	0.011	1.0	0.013	1.00	0.0024	1.00	------	0.4855
**(R)-carv. + LPS**	0.005	0.43	0.012	0.89	0.0015	0.63	0.1810	0.8860

PI/LC: Protein of Interest/Loading Control; **NR:** Normalized Ratio

Supplementary Tables S3

Supplementary raw data to Figure 7A

Nrf2/Lamin B1 ratios and respective p values

	**N1**		**N2**		**N3**		**N4**		**p-value**
	**PI/LC**	**NR**	**PI/LC**	**NR**	**PI/LC**	**NR**	**PI/LC**	**NR**	***vs.* Ctrl**
**Ctrl**	0.005	1.0	0.020	1.0	0.008	1.0	0.007	1.0	------
**(R)-carv. 30 min**	0.010	2.04	0.030	1.48	0.011	1.40	0.015	2.33	0.0586
**(R)-carv. 60 min**	0.023	4.53	0.033	1.63	0.015	1.88	0.041	6.25	0.1640

PI/LC: Protein of Interest/Loading Control; **NR:** Normalized Ratio

Supplementary raw data to Figure 7B

HO-1/β-Tubulin I ratios and respective p values

	**N1**		**N2**		**N3**		**N4**		**p-value**
	**PI/LC**	**NR**	**PI/LC**	**NR**	**PI/LC**	**NR**	**PI/LC**	**NR**	***vs.* Ctrl**
**Ctrl**	0.009	1.0	0.003	1.0	0.033	1.0	0.002	1.0	------
**(R)-carv. 120 µM**	0.036	4.13	0.008	2.53	0.118	3.62	0.001	0.50	0,2497
**(R)-carv. 330 µM**	0.158	18.18	0.017	5.14	0.816	25.07	0.056	22.90	0,0687
**(R)-carv. 665 µM**	0.188	21.59	0.032	9.67	1.418	46.01	0.160	65.00	0,1403

PI/LC: Protein of Interest/Loading Control; **NR:** Normalized Ratio

Supplementary raw data to Figure 7C

Nrf2/Lamin B1 ratios and respective p values

	**N1**		**N2**		**N3**		**p-value**
	**PI/LC**	**NR**	**PI/LC**	**NR**	**PI/LC**	**NR**	***vs.* Ctrl**
**Ctrl**	0.003	1.0	0.0013	1.0	0.0012	1.0	------
**LPS 30 min**	0.007	2.07	0.0007	0.57	0.0027	2.30	0,6546
**LPS 60 min**	0.018	5.64	0.0017	1.28	0.0015	1.26	0,6577
**(R)-carv. + LPS 30 min**	0.025	7.94	0.0080	6.13	0.0039	3.40	0,1451
**(R)-carv. + LPS 60 min**	0.048	15.01	0.0086	6.63	0.0045	3.87	0,3190

PI/LC: Protein of Interest/Loading Control; **NR:** Normalized Ratio

Supplementary raw data to Figure 7D

HO-1/β-Tubulin I ratios and respective p values

	N1		N2		N3		N4		N5		N6		N7		p-value
	**PI/LC**	**NR**	**PI/LC**	**NR**	**PI/LC**	**NR**	**PI/LC**	**NR**	**PI/LC**	**NR**	**PI/LC**	**NR**	**PI/LC**	**NR**	***vs.* Ctrl**
**Ctrl**	0.007	1.0	0.06	1.0	0.003	1.0	0.006	1.0	0.004	1.0	0.058	1.0	0.03	1.0	------
**LPS**	0.055	8.38	0.95	17.29	0.195	58.20	0.036	6.14	0.057	14.24	0.083	1.43	1.07	32.75	0.1258
**(R)-carv. 120 µM + LPS**	0.056	8.50	1.43	25.95	0.218	65.24	0.016	2.75	0.045	11.34	0.069	1.18	0.99	30.26	0.1618
**(R)-carv. 330 µM + LPS**	0.069	10.51	1.52	27.55	0.117	34.91	0.015	2.57	0.054	13.49	0.075	1.29	3.21	98.53	0.2336
**(R)-carv. 665 µM + LPS**	0.126	19.05	1.03	18.72	0.225	67.22	0.021	3.63	0.072	18.15	0.118	2.02	3.76	115.37	0.1990

PI/LC: Protein of Interest/Loading Control; **NR:** Normalized Ratio

Supplementary Figure S1

Uncropped blots shown in **Figure 2A**

p-p38

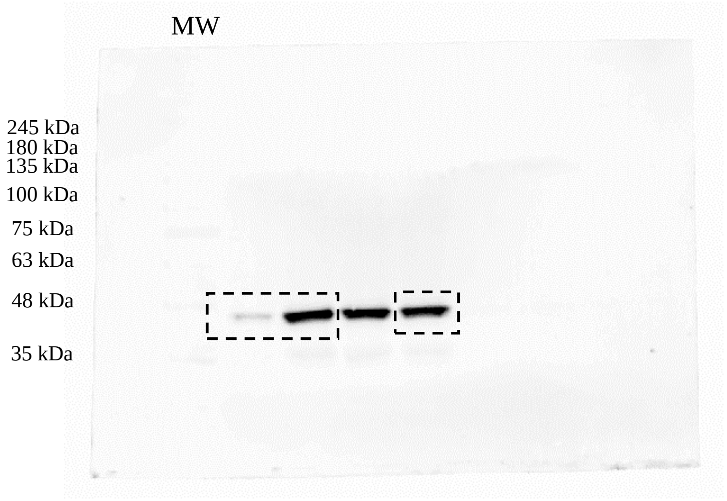

p38

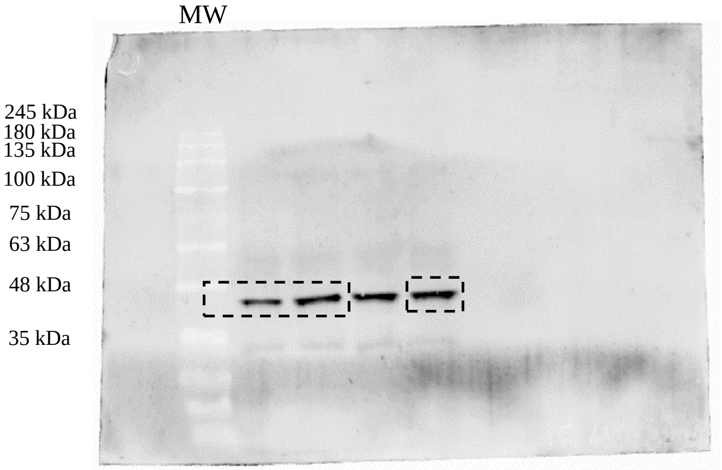

β-Tubulin I

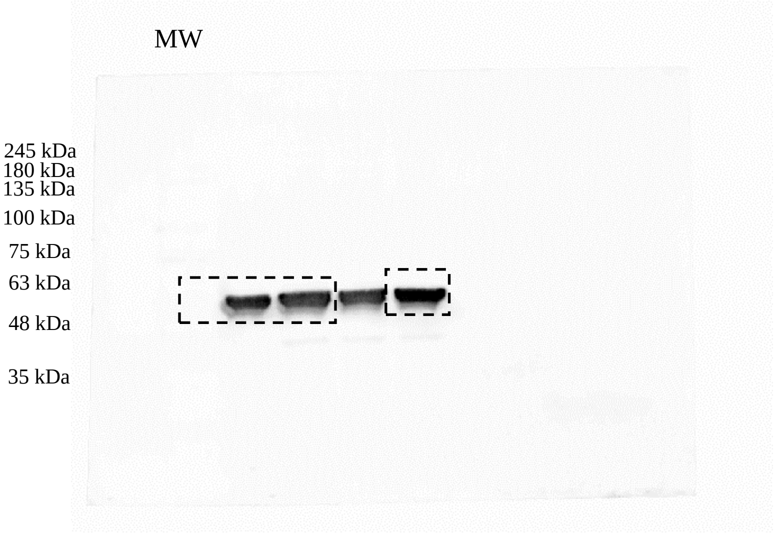

MW: molecular weight marker

Uncropped blots shown in **Figure 2B**

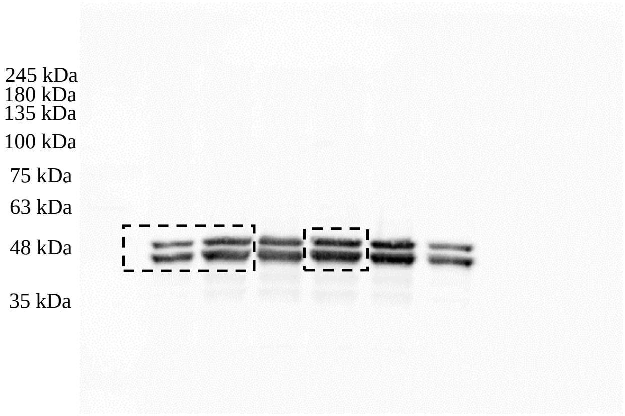

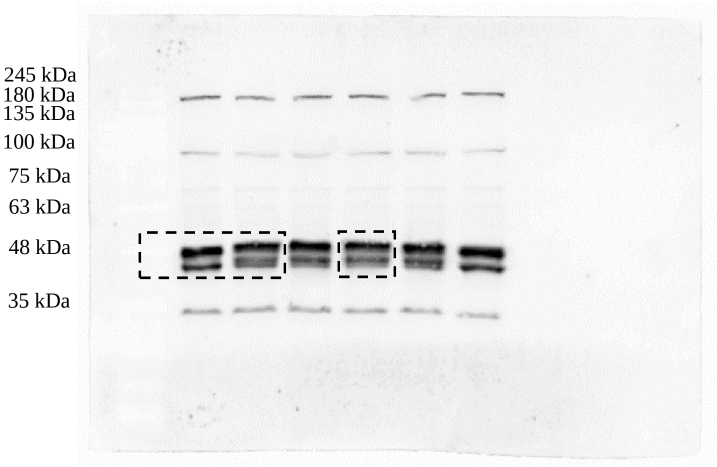

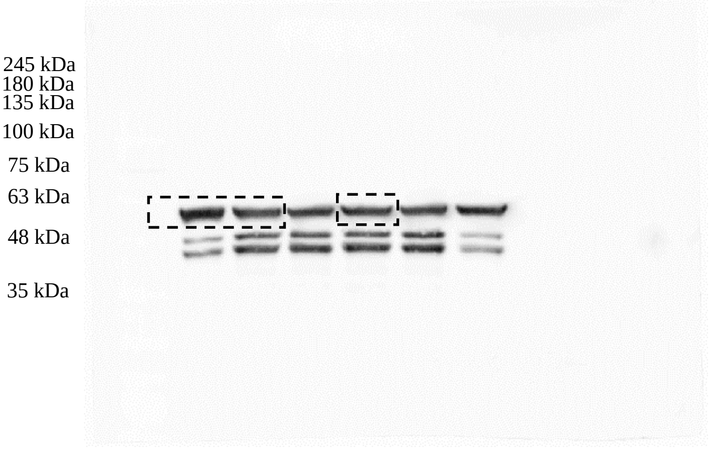

This membrane was probed with the anti-β-Tubulin I antibody without stripping the p-ERK1/2 antibody.

MW: molecular weight marker

Uncropped blots shown in **Figure 2C**

p-JNK

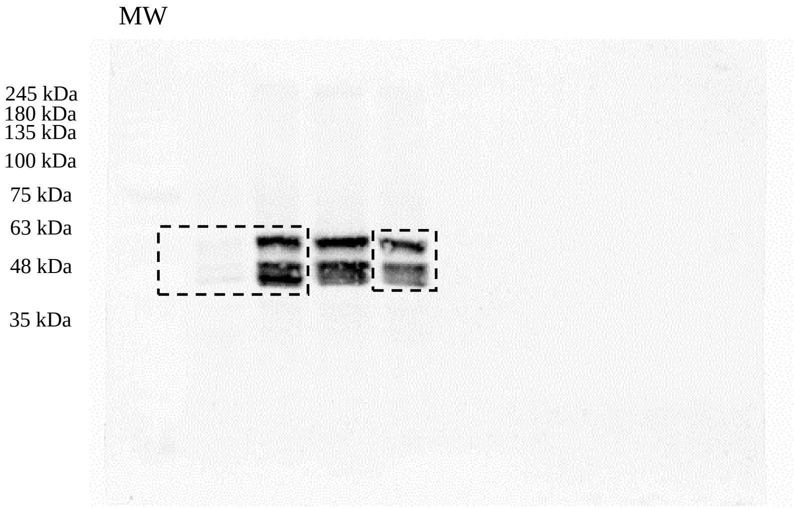

JNK

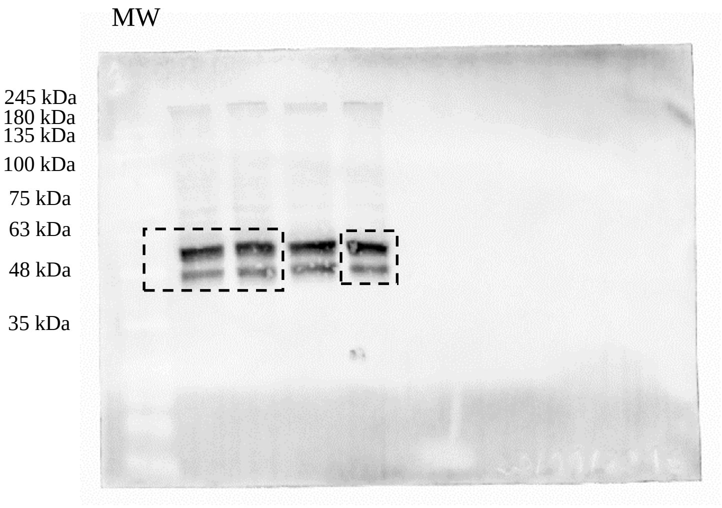

β-Tubulin I

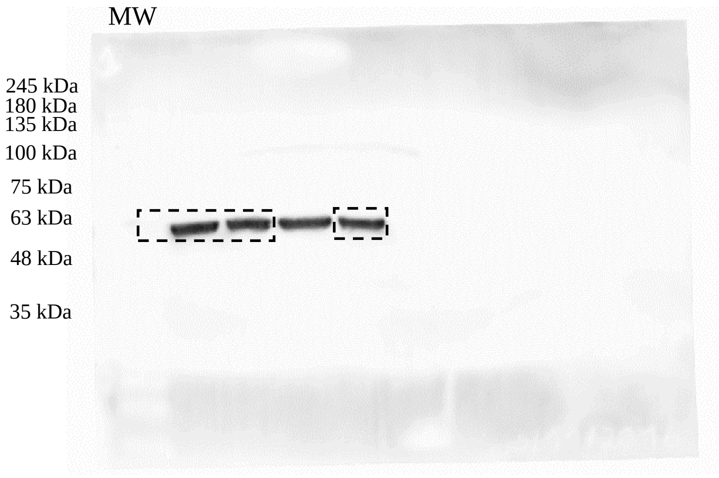

MW: molecular weight marker

Uncropped blots shown in **Figure 3A**

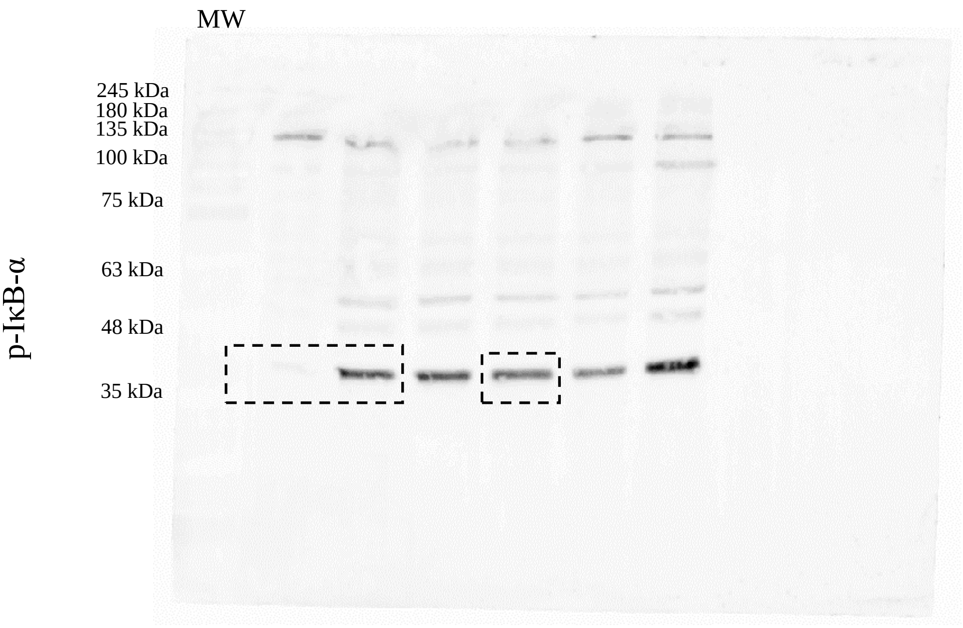

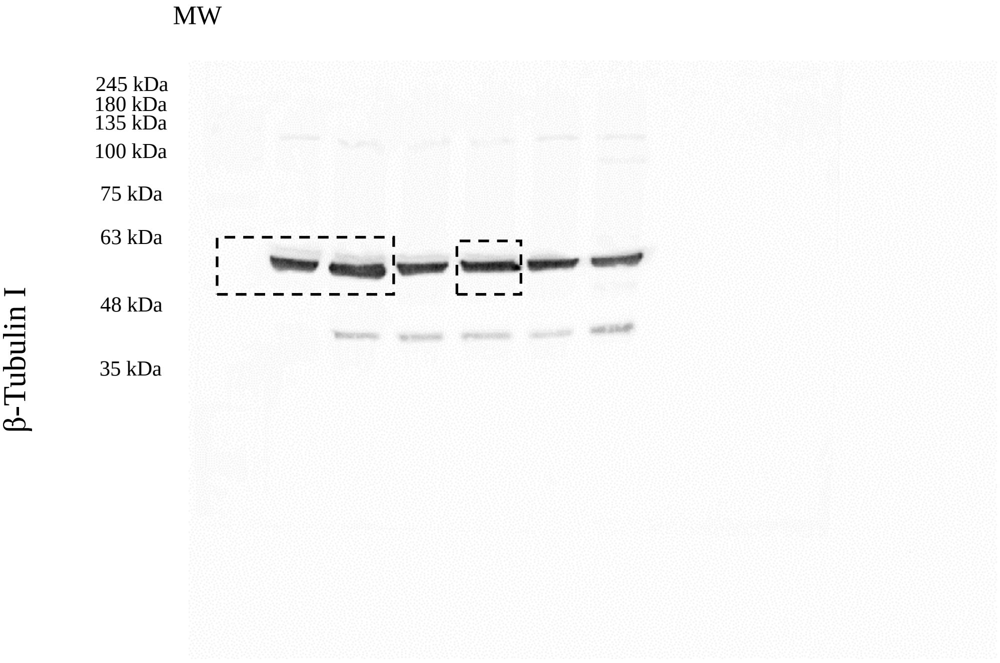

This membrane was probed with the anti-β-Tubulin I antibody without stripping the p-IκB-α antibody.

MW: molecular weight marker

Uncropped blots shown in **Figure 3B**

IκB-α

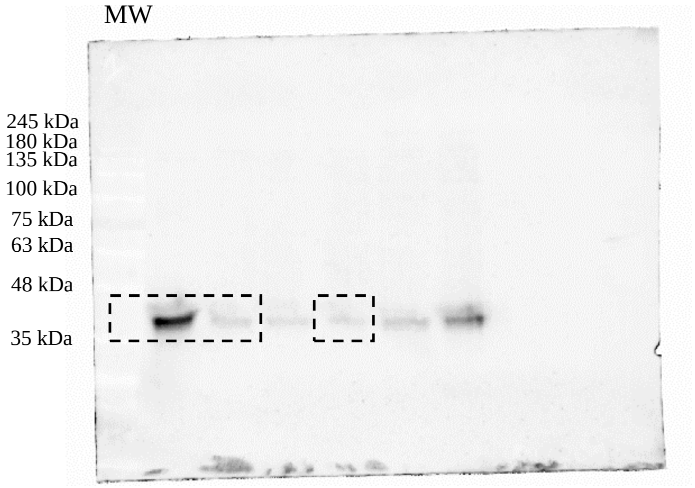

β-Tubulin I

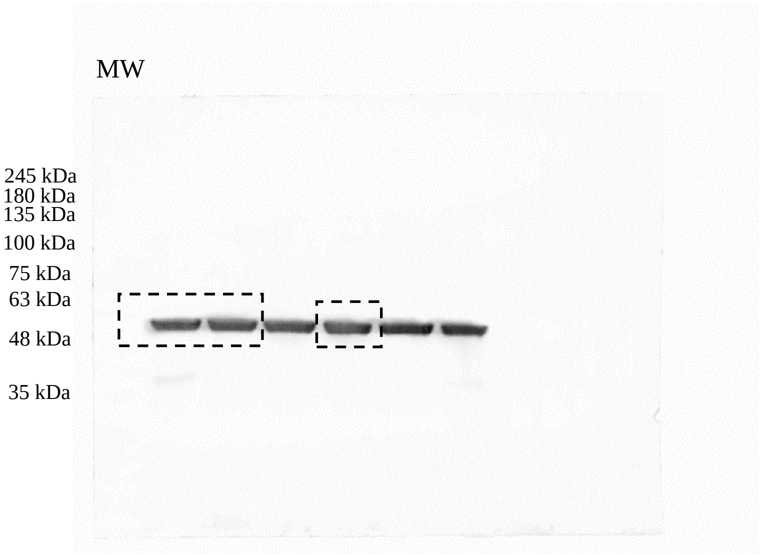

MW: molecular weight marker

Uncropped blots shown in **Figure 4B, left side**

p65

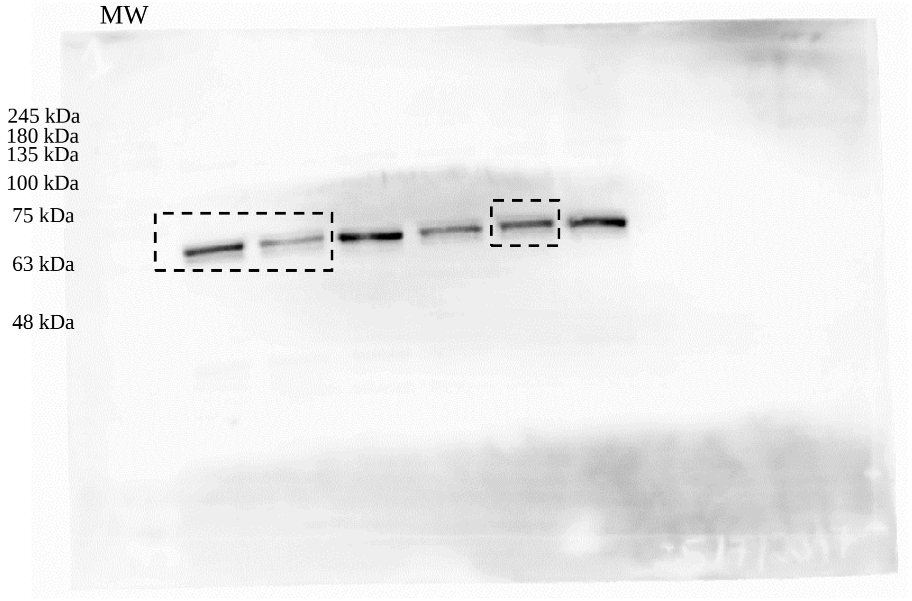

β-Tubulin I

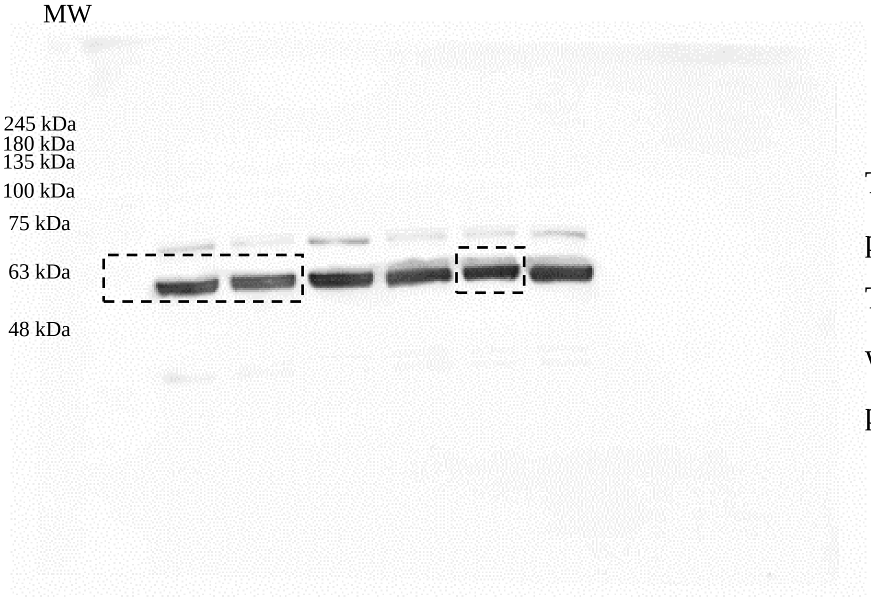

This membrane was probed with the anti-β-Tubulin I antibody without stripping the p65 antibody.

MW: molecular weight marker

Uncropped blots shown in **Figure 4B, right side**

p65

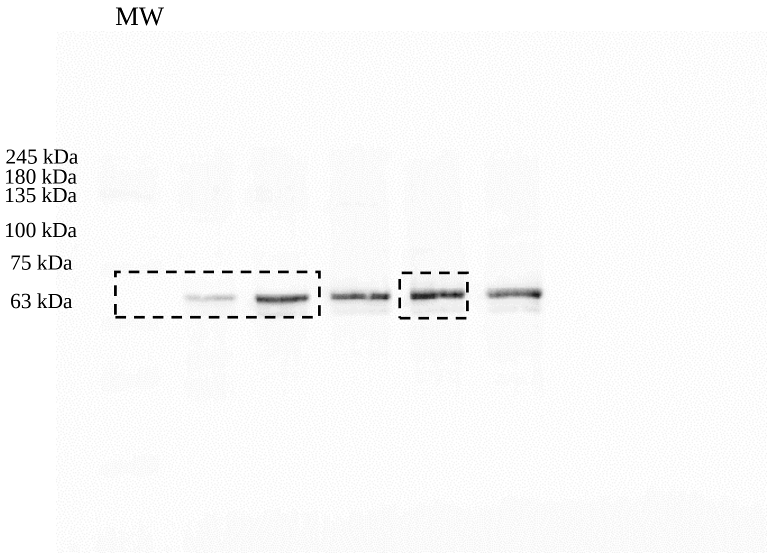

Lamin B1

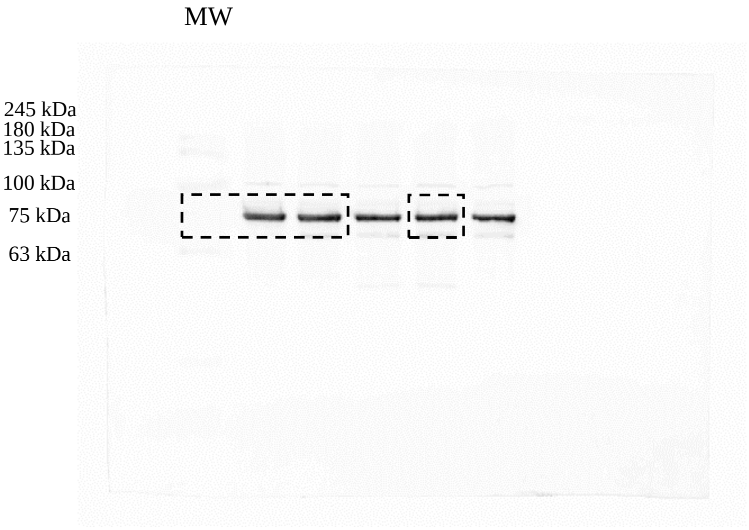

MW: molecular weight marker

Uncropped blots shown in **Figure 4C**

p-p65(Ser536)

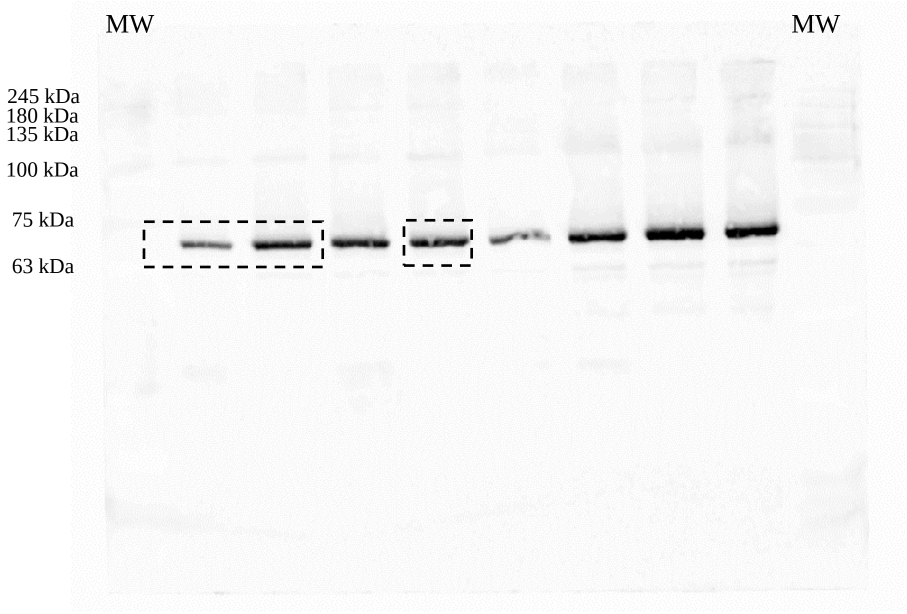

p65

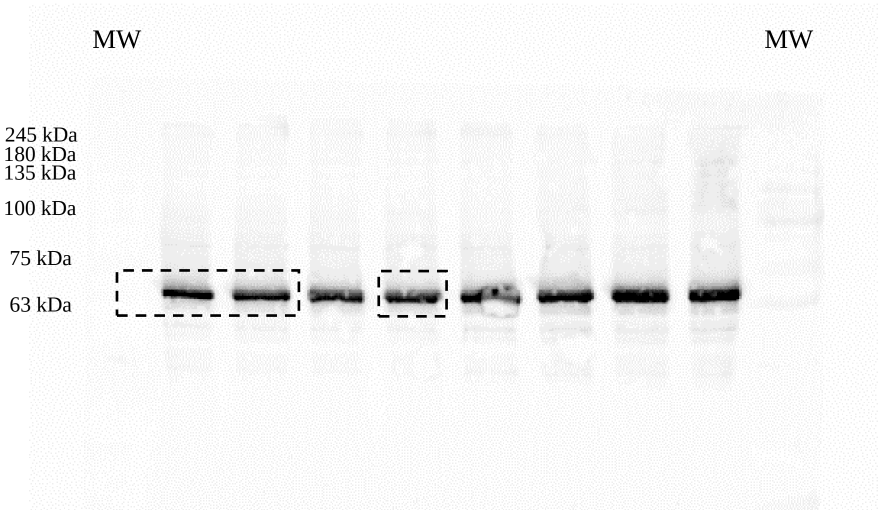

β-Tubulin I

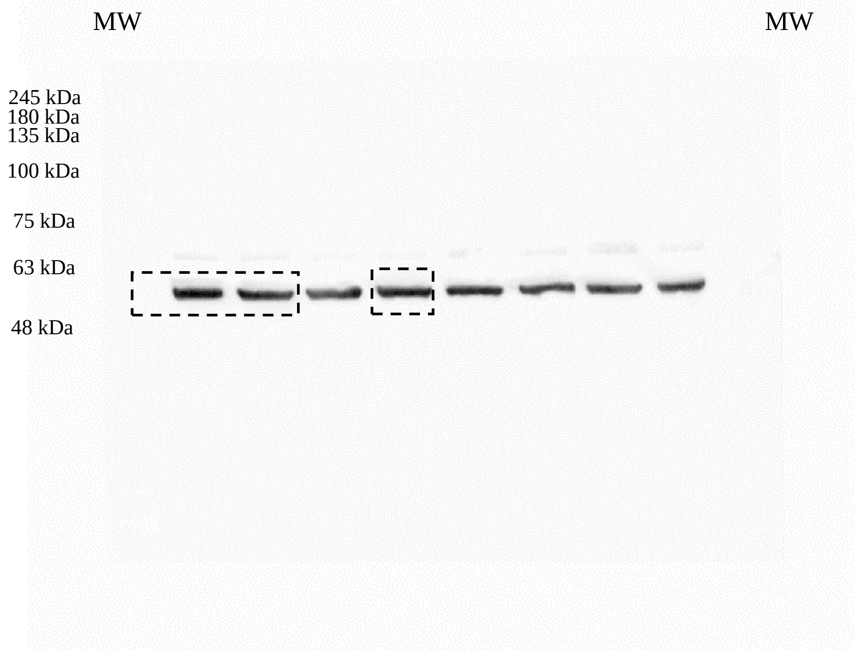

': molecular weight marker

Uncropped blots shown in **Figure 5**

IκB-α

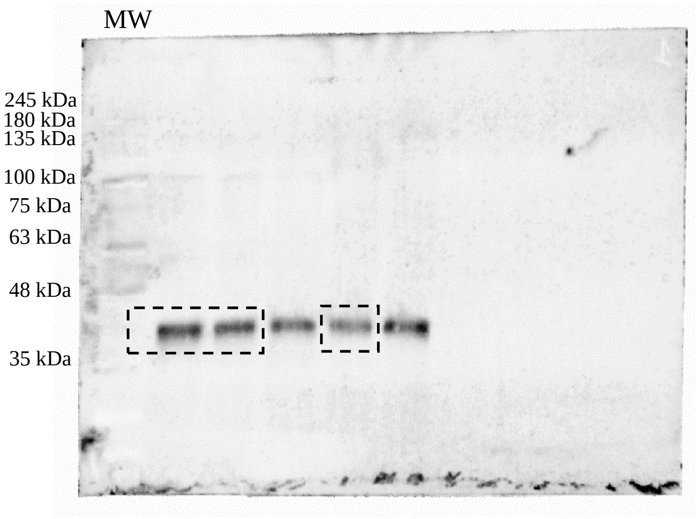

β-Tubulin I

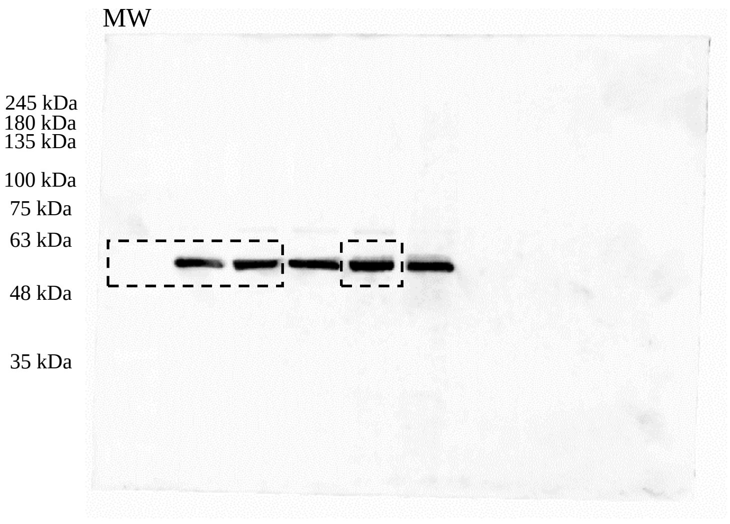

MW: molecular weight marker

Uncropped blots shown in **Figure 6A**

Acp65 (Lys310)

MW

245 kDa
180 kDa
135 kDa
100 kDa
75 kDa
63 kDa
48 kDa

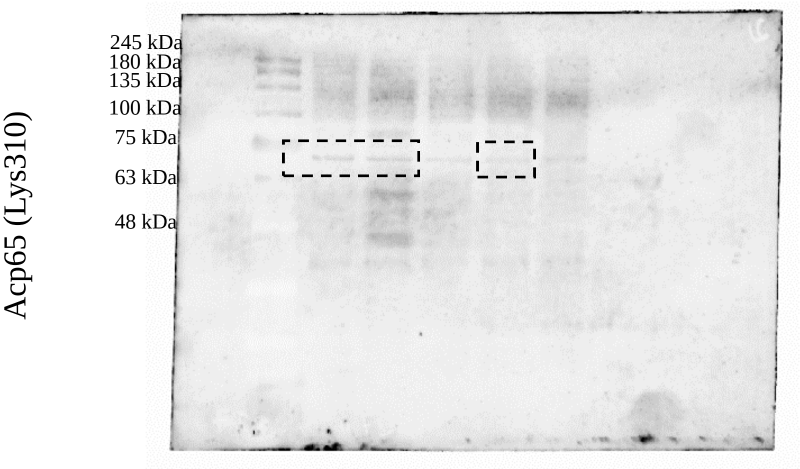

p65

MW

245 kDa
180 kDa
135 kDa
100 kDa
75 kDa
63 kDa
48 kDa

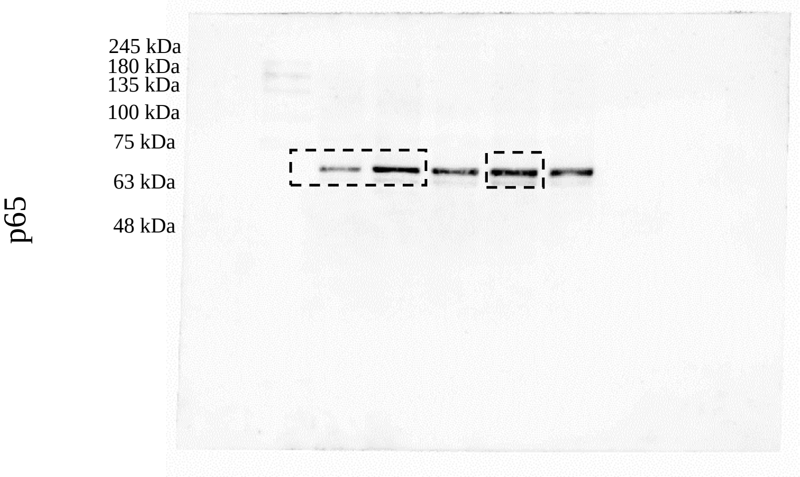

Lamin B1

MW

245 kDa
180 kDa
135 kDa
100 kDa
75 kDa
63 kDa
48 kDa

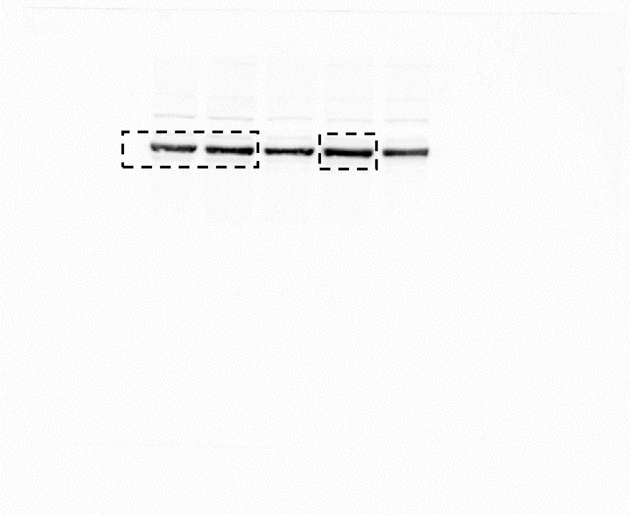

MW: molecular weight marker

Uncropped blots shown in **Figure 6C**

SIRT1

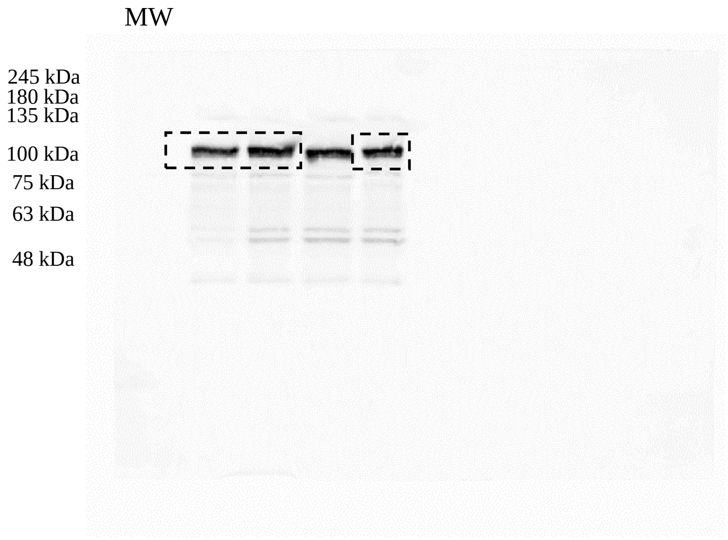

β-Tubulin I

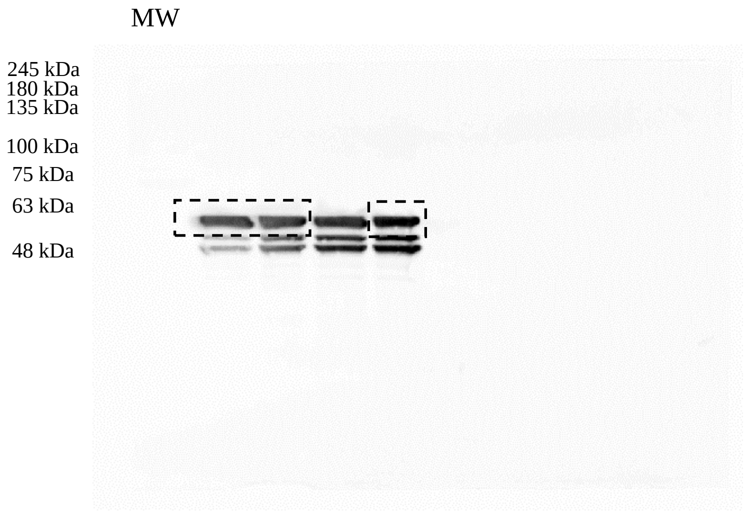

This membrane was probed with the anti-β-Tubulin I antibody without stripping the p-ERK 1/2 antibody.

MW: molecular weight marker

Uncropped blots shown in **Figure 7A**

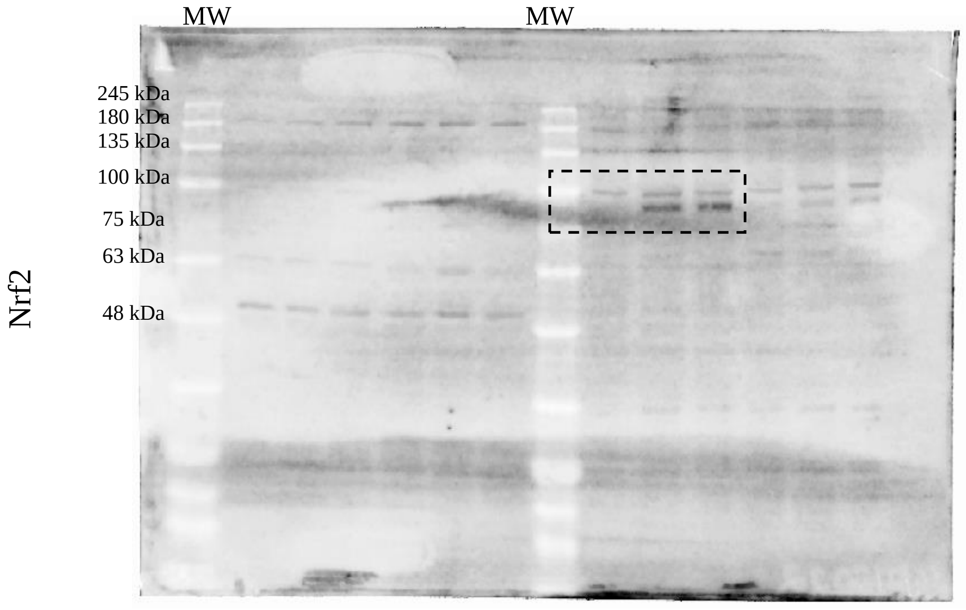

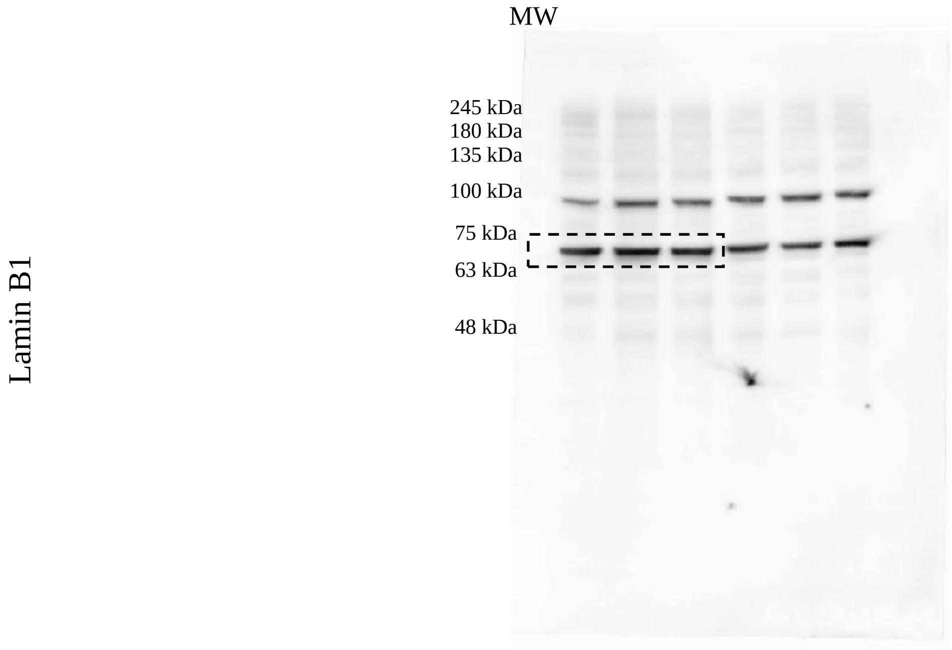

MW: molecular weight marker

Uncropped blots shown in **Figure 7B**

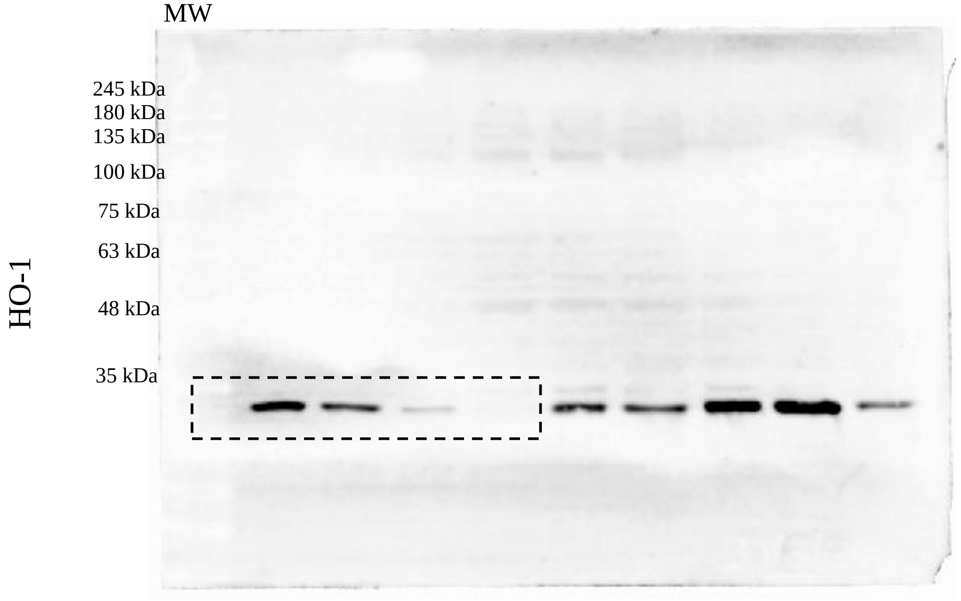

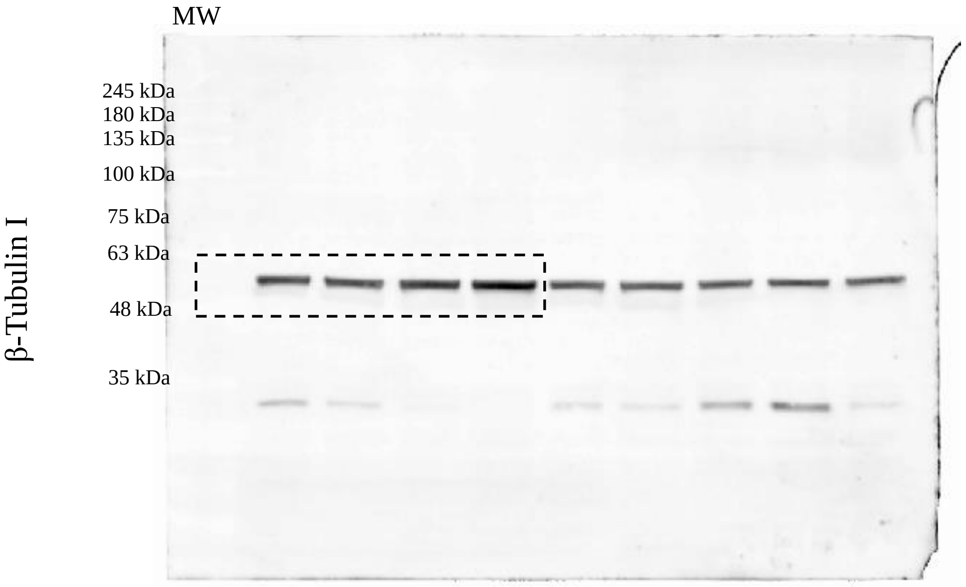

MW: molecular weight marker

Uncropped blots shown in **Figure 7C**

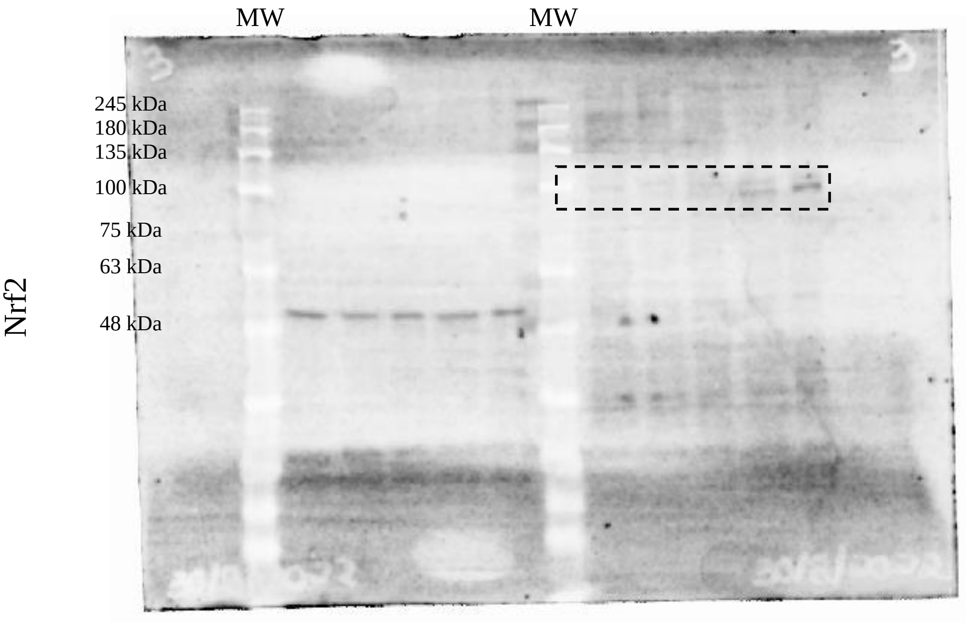

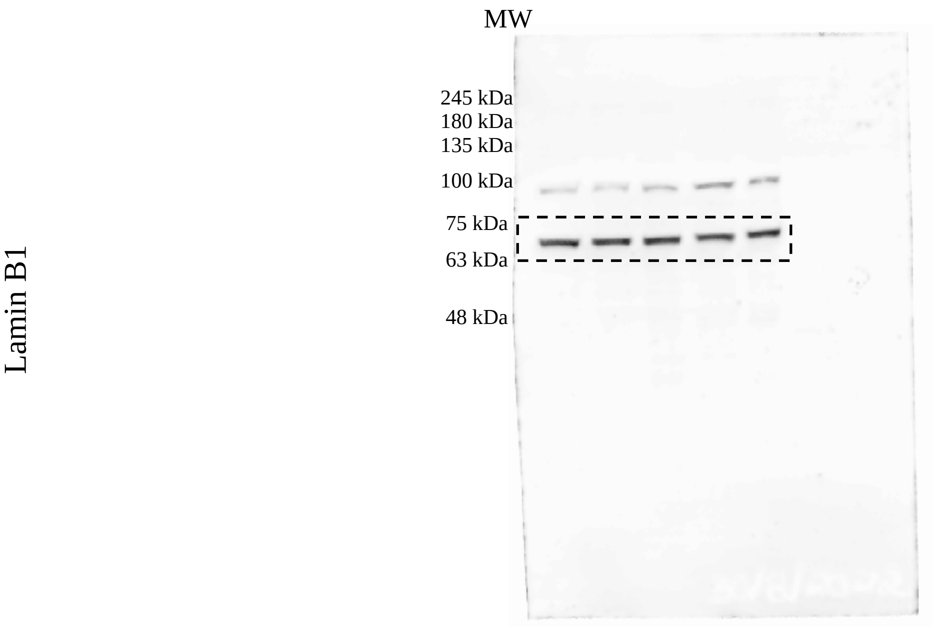

MW: molecular weight marker

Uncropped blots shown in **Figure 7D**

HO-1

MW

245 kDa
180 kDa
135 kDa
100 kDa
75 kDa
63 kDa
48 kDa
35 kDa

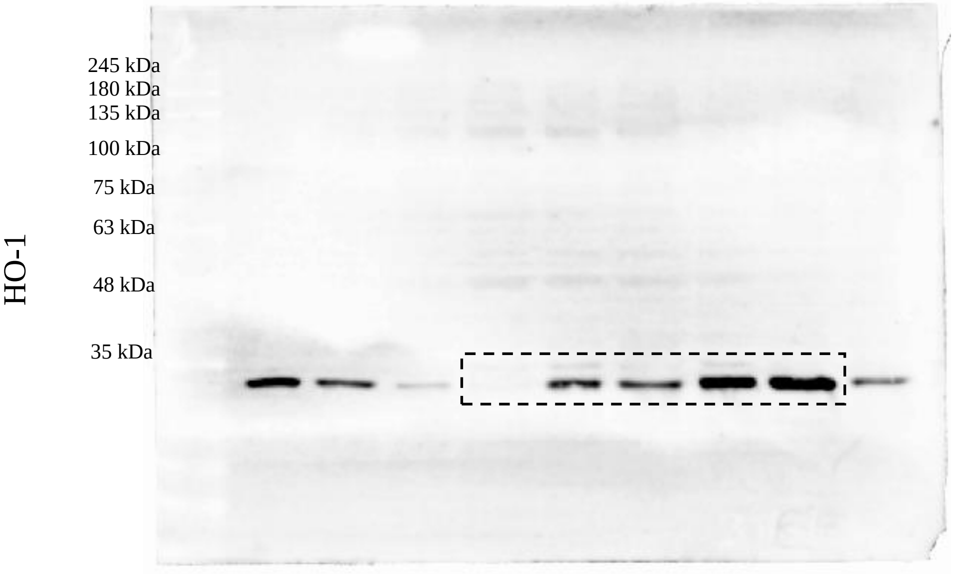

β-Tubulin I

MW

245 kDa
180 kDa
135 kDa
100 kDa
75 kDa
63 kDa
48 kDa
35 kDa

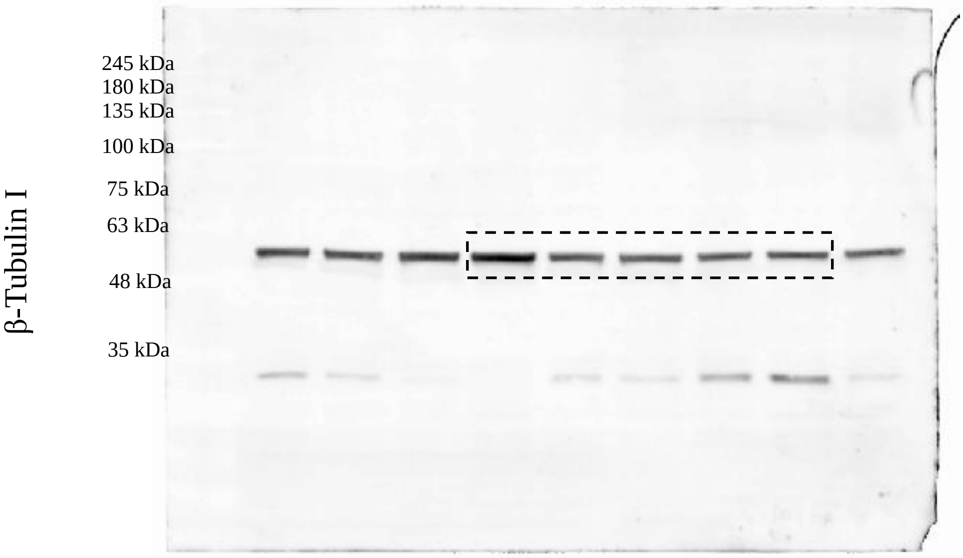

MW: molecular weight marker
